# The Treatment of Backward Displacements of the Uterus and of Prolapsus Uteri by the New Method of Shortening the Round Ligaments

**Published:** 1884-06

**Authors:** 


### Notes on Books.

*The Treatment of Backward Displacements of the Uterus and of Prolapsus Uteri by the new method of Shortening the Round Ligaments.* By W. ALEXANDER, M.D. Pp. 71. London: J. & A. CHURCHILL. 1884.

Two things strike us with regard to this operation: first, that it is a very ingenious one; secondly, that, in spite of the wide publicity given to it, it has been curiously neglected by our leading men in obstetric surgery. Is this apparent neglect only a justifiable distrust? We fear that it is, and that this operation of Dr. Alexander's, like his other one of ligaturing the vertebral arteries for epilepsy, will not command the universal and enthusiastic support of the profession.

It is doubtful if Dr. Alexander's views as to the dynamics of uterine displacements would be accepted by the most eminent gynæcologists; but this we should not lay much stress on if his operative results were favourable. These must speak for themselves. Twenty-two cases are recorded, and success is claimed for all of them. Let us glance at a few of them. Case 1 is for prolapse and cystocele. The prolapse was cured; "the position of the cystocele was scarcely changed." In case 2 "the results are the same as in the preceding case." In case 4 he failed to catch up one ligament properly; but got a good result, except that the patient suffered as before from epilepsy. Case 5 was an epileptic; ligature of both vertebrals failed to cure her, but shortening of the round ligaments for retroversion did. Case 6 was in bed six weeks after operation for prolapse, and was cured. Case 8 was a doubtful success, because of uterine adhesions. Case 9 was more than cured, for she was left "with slight anteversion and prolapse." Cases 12 and 13 had good immediate results, but had not returned to satisfy him as to the permanency of their cure. In case 15 he used a galvanic stem pessary for retroversion and retroflexion during the operative treatment; "but the ligaments had evidently yielded," and at the end the position was improved. She had "fainting fits," which were not improved; but he thinks "that the epilepsy from which she now suffers would not be cured by removal of all the uterine organs." So do we. Case 16 had also a galvanic stem pessary. Case 19, aged 22, with retroversion, was in bed three weeks after operation with a Hodge's pessary in the vagina, which was left in about two months more. Case 20 was a puny woman, "subject to dyspeptic symptoms and miserable forebodings." For retroversion and some prolapse she was operated on on September 6th, and had a stem pessary and a Hodge inserted. On February 20th the uterus was in position; but "she is still puny and dyspeptic, but is improving under the idea that her uterine organs are all right." We have seen how the operation may cure epilepsy; we confess we did not expect it to cure puniness and dyspepsia. "In the next two cases I endeavoured to remove the ovaries by pulling them out in the groins by the round ligaments. The first case shows that where adhesions do not exist it can readily be done; but even then it is inferior to abdominal section. In the second case, the ovaries being adherent, the operation failed."

Supposing that all the others were complete successes, we doubt if each of those we have quoted would be so considered. Looking at the operation as a whole, we cannot fail to be struck with the length of time the patient is under treatment in bed; the amount of suppuration that arises from the wounds; and the frequency with which adjuvant pessaries, intra-uterine or vaginal, are used. Not always, either, is it possible to put the uterus in position; for a ligament may refuse to be shortened, or a uterus may refuse to be straightened.

We think that Dr. Alexander has not done himself or his operation justice in this work. The scientific basis of the operation is not presented with that amount of care and exactitude which the recent abundant labours of original workers on the anatomy of the pelvic floor have rendered possible; and the cases embodying the practical results are not narrated in a way to satisfy the clinician. In spite of this, we believe that Dr. Alexander's operation has a future before it, but that its area ought to be limited to a very circumscribed class of cases. Whether or not it is destined to take its place among the standard operations of surgery, its conception and execution will be a lasting honour to one of the most original and enterprising of our provincial surgeons.

